# Approaching confidentiality at a familial level in genomic medicine: a focus group study with healthcare professionals

**DOI:** 10.1136/bmjopen-2016-012443

**Published:** 2017-02-03

**Authors:** Sandi Dheensa, Angela Fenwick, Anneke Lucassen

**Affiliations:** 1 Faculty of Medicine, Clinical Ethics and Law, University of Southampton, Southampton General Hospital, Southampton, UK; 2 Wessex Clinical Genetics Service, Princess Anne Hospital, University Hospitals Southampton Foundation Trust, Southampton, UK

**Keywords:** MEDICAL ETHICS, GENETICS, QUALITATIVE RESEARCH

## Abstract

**Objectives:**

Clinical genetics guidelines from 2011 conceptualise genetic information as confidential to families, not individuals. The normative consequence of this is that the family's interest is the primary consideration and genetic information is shared unless there are good reasons not to do so. We investigated healthcare professionals' (HCPs') views about, and reasoning around, individual and familial approaches to confidentiality and how such views influenced their practice.

**Method:**

16 focus groups with 80 HCPs working in/with clinical genetics services were analysed, drawing on grounded theory.

**Results:**

Participants raised seven problems with, and arguments against, going beyond the individual approach to confidentiality. These problems fell into two overlapping categories: ‘relationships’ and ‘structures’. Most participants had never considered ways to—or thought it was impossible to—treat familial genetic information and personal information differently. They worried that putting the familial approach into practice could disrupt family dynamics and erode patient trust in the health service. They also thought they had insufficient resources to share information and feared that sharing might change the standard of care and make them more vulnerable to liability.

**Conclusions:**

A familial approach to confidentiality has not been accepted or adopted as a standard, but wider research suggests that some of the problems HCPs perceived are surmountable and sharing in the interest of the family can be achieved. However, further research is needed to explore how personal and familial genetic information can be separated in practice. Our findings are relevant to HCPs across health services who are starting to use genome tests as part of their routine investigations.

**Strengths and limitations of this study**

- Our study is the first to explore, specifically and in detail, approaches to confidentiality and responsibilities to relatives.
- It builds on previous work that has interrogated the familial approach to confidentiality but is novel in that it has explored views about the approach using empirical methods.
- One limitation is that some discussions were around hypothetical issues.
- Although our qualitative approach does not aim for generalisability, our findings are transferable. The details about our research context will enable other healthcare professionals within and outside the UK to determine the extent to which the findings apply to their setting.

## Introduction

Healthcare professionals (HCPs) frequently encounter cases where patients have apparently not told family members about a heritable risk that is relevant to them. In this empirical study, we wish to shed light on two types of these cases: first, when the patient has refused to inform family members or to give consent to allow HCPs to inform on their behalf. Second, a potentially more common problem, where consent is ambiguous: a patient was seen in the past (possibly some years ago) when they agreed to share results with relatives but it becomes evident from a new patient (their relative) that this has not happened.^1^ ^2^ Although outright refusals to share information are reportedly rare,^3^ patients often delay telling relatives or state an intention to tell but for some reason do not.^1^ ^2^ One might consider that here, the HCP faces a conflict of normative duties and values: respecting individual confidentiality and autonomy on one hand and preventing potential harm to a relative on the other.

A view of confidentiality in which genetic information is conceptualised as confidential at the familial rather than the individual level is the ‘joint account’ (which we hereafter refer to as the ‘familial approach’ for clarity).^4–6^ It proposes that potentially familial genetic information should be available to all at-risk family members and so HCPs should consider taking disclosure of this information to relatives as their default starting position. The question, ‘can we share the patient's information?’ is reconsidered as, ‘can we share the familial genetic information with those to whom it might be relevant?’ The normative consequence regarding the two cases is that the conflict of duties is reconceptualised: the interest of all those who might have the inherited finding is the primary consideration and familial genetic information is shared, unless there are good reasons not to do so. Current UK guidelines from the Joint Committee on Medical Genetics (JCMG)^7^ incorporate such a familial approach and encourage HCPs to discuss family relationships, communication of information and the types of information that might be shared at the time of testing. The guidance recommends that the onus might be left with patients to communicate with family in the first instance, but where a HCP realises this has not happened and there is a benefit to a family member to be had, they would not be vetoed from sharing the familial genetic information by lack of explicit consent. Importantly, the HCP would still need to consider the potential harms that might arise from sharing this genetic information.

Beneficence, justice and reciprocity underpin this familial approach. To explain, determining a patient’s risk requires the patient's family history to be taken into account, along with the patient's signs and symptoms. Once the patient's risk status is known, reciprocity towards those relatives who may not have had the opportunity for, but might benefit from, genetic testing, may be appropriate.

Like the JCMG, commentators in the USA have suggested that HCPs should make clear to patients that they will share information and treat the family, not the individual, as their patient.^8^ ^9^ Australian policy about genomic research results is comparable: rather than treating all results as confidential to one person by default, researchers must make clear that the participant cannot prevent the sharing of information that could benefit family members at risk of a serious illness for which treatment is available or pending.^10^ Box 1 features an example of a situation in which a HCP might share just familial genetic information.

Box 1 **Separating individual and familial genetic information in practice**

Patient A is seen at time 1. He has had prostate cancer but also has a family history of breast cancer. A genetic test shows he has a *BRCA2* mutation. Patient B, patient A's sister, is seen at time 2. There is no evidence that she knows about the *BRCA2* mutation but she knows about the family history of breast cancer. Healthcare professionals (HCPs) can tell patient B, ‘we know your family history is in part explained by a *BRCA2* mutation and we can test you for it’. This will not say anything about patient A. If, by any chance, patient B says, ‘do you know this because of my brother?’, the HCP can reply, ‘we know this because of the pattern of cancers in your family’. No confidence of personal information would have been betrayed and nothing about patient A's medical diagnosis shared. This action could be taken if patient A had given consent to share information, if he had refused consent to share, and if his consent was unclear.

### Why explore this now?

Confidentiality and HCPs' responsibility to relatives in genetic medicine is ripe for examination. First, usage of broad approaches to genome analysis, with their greater pick up rate of heritable findings (including primary findings and secondary/incidental findings), are on the increase. A case in point is the UK National Health Service (NHS), which is setting up a genomic medicine service in which patients and their families will be offered whole-exome or whole-genome sequencing as a frontline test across specialties.^11^ Often, primary and secondary/incidental findings will require testing of close relatives to determine their clinical significance. A boom in the number of potentially at-risk relatives identified is thus imminent, and HCPs will need to consider how they negotiate confidentiality and communication of relevant information in this context. Second, the UK has recently heard its first court cases^12^ ^13^ that considered whether a duty of care is owed by HCPs to their patients' relatives. These cases do not shed light on HCPs' perceived professional and/or moral responsibility to *some* relatives, nor whether in *some* cases one could argue that HCPs have such a responsibility, because the courts' arguments focused specifically on the concept of duty of care, and whether this duty would be owed to *all* relatives.

Although many studies have explored patients' experiences of, and barriers to, communication within families,^14^ there is currently no empirical research exploring HCPs' views about confidentiality in clinical genetics. The extent to which UK practice has incorporated guidelines, whether and to what extent the familial approach is accepted and adopted as a standard, and the concerns HCPs have about it, are unclear. Previous research we undertook with patients showed that, to an extent, they supported the familial approach: participants thought genetic information was familial that family members had a ‘right to know’ their risk and that the range of harms justifying disclosure without clear consent was broad. Crucially, they wanted HCPs to tell them about their approach to sharing familial genetic information at the outset.^15^ Our recent systematic review by contrast showed that although HCPs did generally feel a responsibility towards their patients' relatives, they perceived a range of barriers to sharing information.^16^ They thought doing so could violate the patient's or the relative's privacy; found it difficult to define medical benefit and thus whether information warranted disclosure; and perceived a strong obligation to respect the wishes of the patient who explicitly refused to tell their family members about a risk. The findings from this review are tentative: just nine studies were with HCPs and none explored confidentiality and responsibility to relatives specifically; most used basic surveys; almost all focussed on cases where patients explicitly refused to tell their family rather than where consent was ambiguous; and none explored the concept of distinguishing genetic information from personal clinical information.

Our study therefore aimed to elicit HCPs' views about, reasoning around and conceptual understanding of confidentiality; their perceived responsibility to patients' relatives; and the way these considerations related to their practice.

## Methods

### Design

This study was qualitative and drew on grounded theory.^17^ We chose cross-sectional (one-off) focus groups. These were with groups working in the same department where possible to provide an understanding of the real-life context in which HCPs worked and made decisions.^18^

### Recruitment and sampling

A NHS Research Ethics Committee approved the research on 25/02/13 (reference: 13/SC/0041). Between late 2013 and early 2015, we invited HCPs involved in genetic testing to take part by presenting the study aims and protocol at professional meetings and sending emails with attached information sheets to heads of departments for dissemination to colleagues. Sampling was purposive: we tried to recruit from a range of genetic and affiliated services. It is unclear how many potential participants the information reached and thus the number who chose not to participate.

### Sample and data collection

We designed our topic guide (see box 2) based on the existing literature. We also used the familial and individual approaches to confidentiality as our theoretical framework for the topic guide and for analysis. The topic guide was semistructured and piloted in the first group. Focus group discussions centred mainly on patients with risks of cancers and cardiac conditions, for whom there is often an intervention (eg, treatment, risk-reducing options or surveillance) and thus where communication with relatives is most urgent. SD, a research fellow with a doctorate in health sciences and several years' experience of qualitative research in the field, conducted all focus groups alone. SD audio-recorded the discussions which lasted ∼1 hour, and took field notes of non-verbal aspects of communication, such as nodding. Participants had no previous relationship with her. Sixteen focus groups with 80 HCPs from across the UK were conducted, either in clinical departments or during professional meetings. There were participants from 14 of the 24 UK genetic services. Table 1 details participants' professions.

Box 2 **Topic guide**

Introduction

- What is your role?
- What kinds of patients do you see? How many per week?
- What other departments do you work with?

Confidentiality

Is confidentiality important in the area of medicine that you are working in?

- Why do you think it is important from a patient's point of view?
- And why from a healthcare professionals' (HCPs') perspective?

What aspects of the medical consultation should be kept confidential?

- Probe about personal versus familial genetic information
- Probe about confidentiality in genetic medicine versus other areas of medicine

Are there guidance documents or protocols you follow for confidentiality?

- What do they say?
- Are they widely read?
- Do people agree with them?

Consent

What does/should the consent process involve when a person has a genetic test?

- Is there an official consent process in your department?
- How, if at all, do you talk about the limits of confidentiality in the consent process?
- What do you consider these limits to be?

Information sharing

Regarding genetic test results, who do you think should tell the result to at-risk relatives?

- Probe: the person themselves, a HCP, general practitioner, etc.
- Probe: advantages and disadvantages of each.

Have you ever had experience of a patient telling you they were not going to inform their family of risk? Or a patient who you were not sure had told?

- Probe for details.

To what extent do you feel like you have a responsibility to ensure patients' family members know their risk?

- What, if any, limits does this responsibility have?

The future

Regarding these issues, do you feel like you have enough support and training?

Whom do you talk to about ethical issues?

What are your main concerns, if you have any?

Do you have some other things you would like to raise?

**Table 1:** Details of focus group participants

Profession	Number
Genetic counsellors	37
Clinical scientists (molecular/cytogenetics)	16
Consultants in clinical genetics	8
Registrars (trainees) in clinical genetics	8
Nurses working with a genetics team	4
Fetal medicine professionals	4
Family history coordinators	2
Nephrologist	1
Total	80

FG5, 6 and 7 comprised of participants from different departments. The rest worked in the same department.

### Data analysis

After the first focus group, SD, AL and AF began coding and thematically analysing the data, drawing on elements of grounded theory, such as constant comparison.^17^ We independently interrogated transcripts and then discussed the emerging codes and themes together. The topic guide was adapted over time to pursue emerging lines of inquiry. We ceased recruitment and data collection when we began to approach saturation.

## Findings

### General outline: the conventional steps for facilitating communication

**Figure 1:**
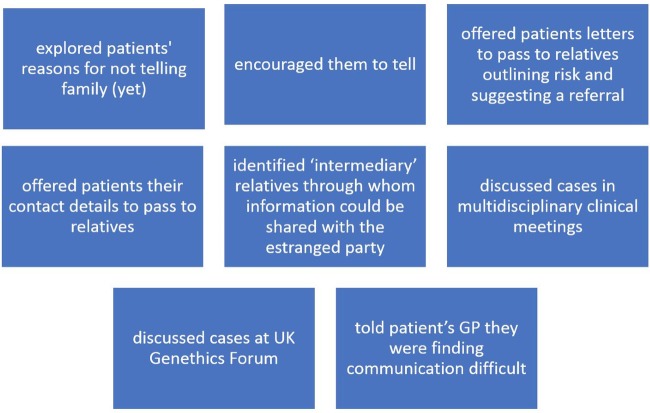
Healthcare professionals’ conventional ways of dealing with non-disclosure.

Participants questioned their responsibility to their patients' relative(s) when consent was ambiguous or when a patient had refused consent to share information. Although participants claimed they rarely encountered the latter situation, they mentioned around 30 different cases where a relative had not been told. Furthermore, they recognised that their awareness might be limited by patients who said they would tell family members but did not actually do so. When a patient refused consent or was reluctant to share information, they had several conventional strategies to encourage disclosure, outlined in figure 1, including discussing cases at the UK Genethics Forum.^19^ No one reported contacting relatives or their general practitioners (GPs) directly, checking databases to find relatives' details or checking family files to see if they had been referred—all of which are strategies that have been used in intervention studies.^20–24^ A consideration when deciding what to do was whether the relative was also their patient or a patient of another genetics service, or whether the relative had not been referred for genetic advice at all. Participants said they had gone, or would go, beyond these conventional steps only in exceptional situations, for example, if there was a child at risk of an early-onset, serious and treatable condition.

**Figure 2:**
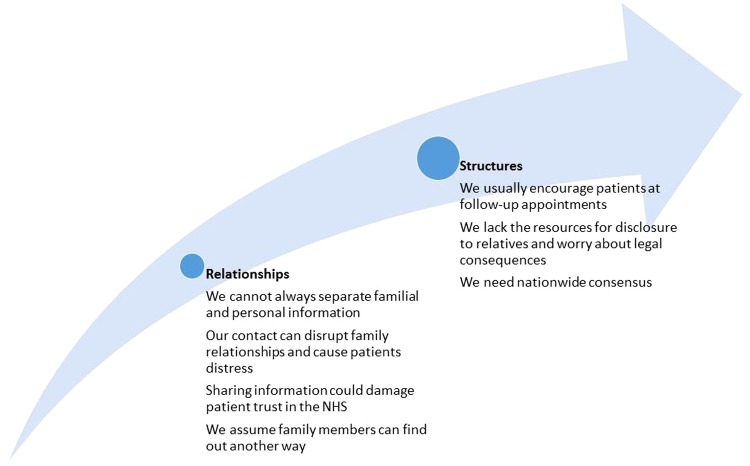
Themes—each corresponds to an argument against using the familial approach.

Rather, they perceived several problems with taking a familial approach to confidentiality, which fell into two overlapping themes and subthemes, summarised in figure 2. The first comprised of concerns about relationships between family members as well as between HCPs and patients. The second pertained to the structure of the health service, comprising practical, legal and ethical considerations about the distribution of resources.

### Relationships

#### We cannot always separate familial and personal information

Some participants considered genetic information familial, but thought disentangling familial information from the patient's personal clinical information was not always possible. Others had not considered whether there was a difference between personal and familial information. They thought that even if they told relatives (directly or via a HCP) that there was a risk in the family, it might point to a particular person who could then claim that their confidence had been breached. Many argued this was more likely than not as they were ‘dealing with rare conditions and it is quite a small world’ (FG14P4). Participants reported that some of their patients apparently felt their relatives had no ‘right to know’ (FG12P2) what they considered ‘their’ diagnosis, even if the diagnosis implied an inheritance that had relevance for these relatives too. As their HCPs', they thought they had to respect this view.

Only some participants talked about personal and familial genetic information as being separable. For example, one participant recalled a case where a new patient concerned about his risk for a familial cancer syndrome had an ‘inkling’ about where distant affected relatives lived. The participant found an old report about one of these relatives where consent was unclear, and shared just the necessary information:

FG10P3: I got her report…he hardly has any contact with that family member. He had an inkling who it was. He didn't know what kind of cancer she'd had [or] anything. He wasn't particularly curious either. All I said to him was, ‘I've done some digging and I've found some information and we have a test we can offer you’. But I completely stepped over the issue of who it was, and what cancer they had.

Along these lines, other participants said that if a HCP made a considered decision to share some information carefully, for example, familial genetic information via a GP, it would not count as a breach:

FG14P6: If you make a decision to write to a [relative's] GP or go down these different aisles of trying to get the information to people, I think breach isn't quite the right term to use, because you're carefully considering the confidentiality in the situation and there's different layers. You're carefully considering ‘yes, actually we think it's appropriate to go to the GP in this situation because the risk of harm [is] high enough’.

A few participants said they would not feel it was a breach of confidentiality on their part if the relative inferred the patient's identity, as long as they themselves had not included any identifying information. However, the potential for inference made many others reluctant to share familial genetic information. Participants felt more compelled to do so if the relative had been referred to them, because they then had a duty of care to both parties, but even this was tricky work, with many talking about how ‘careful’ they had to be.

#### Our contact can disrupt family relationships and cause distress

Family relationships were a key consideration for participants when thinking about sharing information. One reason was that, in their experience, patients who had not (yet) shared information with relatives often had poor relationships with them. Some were anxious that relatives would judge them or were worried about what relatives would do upon learning the information (eg, a previous patient of FG3P1, who was diagnosed with haemophilia, apparently refused to tell her pregnant sister about the possibly familial risk in case she terminated the pregnancy). Participants felt unable to ascertain these ‘intricacies’ of family dynamics and were uncomfortable with the uncertain, potentially ‘enormous’ (FG16P1), and exacerbating impact sharing genetic information could have. Coupled with their worry about separating personal and familial genetic information, this concern led them to err away from sharing information:

FG16P3: [It] really does make me question what responsibility you have and what a mess you could make of that family by sharing that information…we just don't know: you could cause much more psychological damage by disclosing. But you're the keeper of this really difficult piece of information.

 FG16P1: An unknowable risk isn't it, the damage that you could cause by disclosing.

Regarding direct contact with relatives, participants thought it would be more of a shock and more invasive of privacy if a HCP, as opposed to the patient, made contact. They were moreover apprehensive about violating the relative's ‘right not to know’ and said that although a patient would also violate this right if they communicated the information, it was ‘less okay’ for HCPs violate it (FG14P6). Interestingly, participants were reluctant to contact relatives even in situations where a patient had specifically asked them to, as they thought the information would be better received if coming from a family member.

A few participants in FG14 criticised some of their own arguments here, and thought they perhaps should share familial genetic information, despite their anxieties about family dynamics. They considered it a double standard to refuse to invade the privacy of their patient's relative, given that it was standard practice to ask patients to invade the privacy of their relatives (with whom they might not have a close relationship) and indeed forgo their own privacy (in that if the patient shared, the relative would likely realise that they had had the test).

#### Sharing information could damage patient trust in the NHS

A minority of participants pointed out that by not sharing familial genetic information, a relative could come to harm or die, for example from a preventable cancer, and this might outweigh the potential harms of breaching a patient's confidentiality. However, others thought it could damage a patient's trust in the health service if a HCP shared even familial genetic information, which weighed heavily in the balance against doing so:

FG14P3: Ultimately, I’d worry about keeping [my patient's] confidentiality.

 FG14P5: Yeah, but not to the detriment of other people! It's difficult when you stop to philosophise about that: where does our responsibility lie? Does it lie with the family or does it lie with just that patient? It's a very difficult moral issue. There's people out there who could die because you're not telling them, because you're worried about someone getting in trouble with their sister. Which is more important? It's difficult isn't it?

 FG14P3: You don't want no one ever to come forward to see a genetics department.

 FG14P5: They'd feel worried that information will be…

 FG14P3: It's always going to be shared with everyone.

This concern about trust was underpinned by participants feeling that it would not be fair for them to share information when they had not been explicit from the outset that they could. Indeed, an important finding was that while participants considered it a vital part of their role to highlight familial implications, they did not always do so in the first appointment, thinking it important to instead give the patient time to understand, cope with and adjust to their risk or diagnosis. They thought that sharing familial genetic information without having been explicit about it at the outset would violate an implicit social contract and this could in turn damage the relationship between them:

FG10P1: We say to the patient, ‘it's your responsibility to cascade this information in your family. We take no part in that.’ But when they say they're not going to do it, and we say ‘we're going to step in and do it for you’, that's quite tricky.

 FG10P5: Also, you say everything you tell is confidential.

 FG10P2: apart from (!)

 FG10P1: It's a bit mean for those patients to come through thinking everything is going to be confidential, and then for that to backfire on them. They're coming thinking, ‘this is my information’.

Notably, the focus group discussions made some participants reflect on their usual consent practices and question whether they should ask patients to agree to sharing before testing them.

#### We assume family members can find out another way

When patients had not yet told relatives about a risk, participants made hopeful claims that the news would probably be communicated throughout the family eventually. Given this possibility, they did not feel wholly obliged to intervene:

FG8P2: Maybe those [relatives] will find out another way, because they don't often fall out with their whole family. It's often specific people. I've certainly had people come to clinic who have found out about the mutation through some circuitous way.

 FG8P3: Yeah, we often get uncoordinated referrals, in a sense that family members will be referred separately, so often they don't know that they're simultaneously all being seen. Like you said, the news does go round in some way or the other.

Participants also considered the possibility that if given ‘dear relative’ letters, patients who were reluctant or refusing to share information, or to give consent for HCPs to share, would eventually pass them letters on. They moreover thought that if a patient did not share information, an at-risk relative might know their family history and request independent advice from a genetics service anyway.

Other participants explicitly acknowledged that these were all assumptions, and cited several examples where family members had in fact not learnt about their risk through other means for many years. And in some families, relatives had developed a cancer that could have been prevented:

FG14P4: I'm seeing a chap on Thursday. I saw his mum in about 2002 and said your son can be tested, and he's just rocked up now, 12 years later, for a pre-symptomatic test, and he's got the mutation. He could have known that a long time ago.

### Structural issues

#### We usually encourage patients at follow-up appointments

Participants said that in the past, they had taken for granted that they would have follow-up appointments with patients to discuss familial communication. Participants thought these appointments were important to help patients understand their own and their relatives' risks, and to facilitate communication:

FG5P3: The more common reason for non-disclosure is that the penny hasn’t dropped. You think you’ve explained it, they nod at you nicely, [yet] they’re like, ‘what about cousin Sue?’ And you’re thinking, ‘we've done this a thousand times!’ We can’t assume that because it’s easy for us [to understand genetics] it's easy for them. It takes ages.

As several groups discussed, follow-ups were becoming less possible due to health service constraints. Ironically, these cutbacks were happening concurrent to the increasing use of genome tests, which, as discussed earlier, produce results of a greater number and complexity:

FG5P2: It's going to become far more prevalent in our practice as genetic counsellors to encourage people to share information and [think about] how to persuade them to do it. So [genomic medicine] is changing our role, isn’t it? Yet at the same time, we have less contact on the whole with patients than we used to have. And the relationship has changed. So I think it’s very, very challenging.

FG5P1: It's getting harder and harder to discuss communication issues and do the bread-and-butter counsellor stuff a lot of us came into the profession to do, because there's the pressure of seeing more and more people with fewer and fewer resources.

A related problem was that where follow-up appointments were available, patients did not always use them, did not ‘know their way through the NHS’ (FG14P1), or know that ‘the door is open and they can come back if they want’ (FG16P1). Therefore, patients who had not told relatives about a risk sometimes had only one contact with HCPs. Participants realised they could make ways of accessing help clearer. That participants had this concern indicated their preference for building a relationship with the patient and encouraging them to share information themselves.

#### We lack the resources for disclosure to relatives and worry about legal consequences

Participants perceived constrained resources (ie, time, staff, funding, methods of finding contact details) a major barrier to doing anything other than leaving communication entirely up to patients:

FG4P1: There are probably people that we don't contact whom we could, if we had more resources, who could benefit from it. It's always going to lead to some sort of inequality. That is a source of some anxiety in terms of whether we've done the best we can for the wider family and whether we have a duty to do the best we can for the wider family. The best we can do at the moment is generally giving information to the person in front of us. Everything is done through that person. But if our services were configured in a different way, that might not be the limit of what we could offer.

When asked what they would do if, hypothetically, they did have the necessary resources, one response was that they would still be uneasy about contacting at-risk relatives who had not been referred to their service. Sharing familial genetic information could leave them liable for invading the relative's privacy and breaching a patient's confidentiality, but not sharing it, despite having the resources to do so, could leave them liable for negligence. Inadequacy of resources was thus a defence against culpability for this wider duty:

FG14P8: No-one's won a case in America yet which is good. The moment you start saying it is our job to send these letters out, then we've accepted that this is our responsibility. The culpability is a [worry].

Another area of trepidation was where they would be expected to draw the limits of familial contact. Once they contacted one patient, they argued, they might be seen as having a legal ‘duty to warn’ all at-risk relatives—a duty they considered would be impossible to fulfil:

FG14P8: If you accept some of [the responsibility], are you accepting more of it?

FG16P1: [It would] set a very difficult precedent. To what lengths do you go to make sure that information's been passed on, and how do you assess that? Logistically, it would be almost impossible to check out all the relatives at risk [have] been informed. We are miles away from having any kind of infrastructure that would support that.

FG8P1: Where would you ever stop then? How many people would you contact? How far would you go in the family?

One worry here was about opportunity cost—the time spent tracing a relative's GP for example, would mean less time for seeing new patients.

#### We need nationwide consensus

Participants' apprehensions about resources and liability were underpinned by the perception that taking responsibility for relatives of patients would be a substantial shift in genetics practice, because keeping individual patient confidence was the norm, and communicating with relatives, or their GPs, was not yet ‘culturally acceptable’ (FG15P5).

Many participants said they were aware of JCMG guidelines and the familial approach to confidentiality advocated within, but thought the guidelines had not shifted practice. Some desired guidelines with clearer protocols. Some moreover argued that they would be more comfortable taking the sharing of familial genetic information as a default position if doing so was widespread practice, where there would be safety in numbers, better protection from litigation, and equality in care for patients and families nationwide:

FG15P5: [Any change] would have to be a national one, so that everybody could sign up to the same process [with] guidelines that patients could read. Practice could [then] change in the direction of us saying to people before they had a test, ‘part of having a genetic test is the willingness [to communicate]’. But you'd need a background of cultural acceptability. I don't think it could be down to individual practitioners, because different [families, and] even members of the same family might be treated very differently.

FG5P3: We do try to help, [but] there's no protocol for it, there's no standard for how you go about it, even though it [telling relatives] is the right thing to do. It's very hard. It goes wrong in ways you can't anticipate. You're leaving yourself very open as well, and you think, ‘am I interfering with things I shouldn't be interfering with?’

Participants at the same time perceived guidelines to have limited utility, because they typically do not cover every situation and require professional judgement, which can be difficult to make. Producing a more detailed guideline was not deemed a simple solution, particularly since the ‘standard way of reaching a decision’ in difficult situations was through discussion with peers rather than deference to guidance (FG1P1).

## Discussion

Although a few had shared familial genetic information from one individual's test to benefit relatives, participants in our study took an individual approach to confidentiality in general. Akin to the findings of our systematic review,^16^ participants, although perceiving that they might have some responsibility to relatives, identified several reasons for not acting on this responsibility. One set of reasons related to concerns about relationships between family members, and between themselves and their patients. More specifically, they thought that if a patient had not told a family member about a risk, there might be reasons for this relating to poor family dynamics, and divulging information could exacerbate these, could cause distress and could cause patients to lose trust in the NHS. This first concern echoes that of genetics professionals in research from the USA:^25^ ^26^ they considered patient-relative relationships and patients' emotional reactions to be two of the most influential considerations in their decisions about whether to disclose information. These are important concerns, but notably, research has shown that when patients do not share information, there can be outcomes such as disappointment and resentment between family members, which can reduce family cohesion and well-being.^27–31^ HCPs not sharing information could equally be worse for trust in the health service, because relatives who develop a preventable cancer, where HCPs had known about the risk but had chosen not to act, might question their practices.^32^

Two issues that underpinned arguments about relationships in our study were that, first, most HCPs had not considered whether familial genetic information and personal information could be treated differently, or did not think they could be, and so worried that sharing familial genetic information would result in their being blamed for sharing *personal* information. Second, our participants did not always discuss sharing with family members at the outset of their interactions with patients, and thought sharing information later down the line would jeopardise trust and perceived transparency. Indeed, research about sharing medical information in another NHS context—the UK's care.data venture—shows that while people value appropriate and responsible information-sharing—that which could contribute to a ‘common good’, a lack of transparency about what information exactly is shared, with whom, and for what reason, has led to mistrust in the health service.^33^

Relating to our second theme, participants worried that sharing information would be a resource-intensive activity. They believed this because they thought taking on a narrow responsibility for some relatives could change the standard of care and lead to them having to take responsibility for more relatives. Echoing this finding, research with HCPs regarding recent French legislation, which requires HCPs to offer to write to relatives about their possible genetic risk, showed that participants were apprehensive about what this legislation would mean for their responsibility to relatives. It was not clear to them who counts as family, to whom they would have a responsibility or how this responsibility could be properly discharged.^34^ These findings suggest that definitions need to be clearer. Our participants also argued that if the standard of care were to change, they could be liable for not sharing information with all at-risk family members *as well as* for breaching individual confidentiality. This may be due to a perceived ‘litigation culture’ in the health service.

Participants thought that if they were to share familial genetic information by default, all HCPs should do so, as part of a consensus shift in practice. This point was interesting, because with their guidelines, the JCMG^7^ intended to change practice in this way—that is, encourage HCPs to discuss sharing of familial genetic information from the outset and make clear why such sharing was needed and that such sharing could happen. It may be that HCPs were unsure how to implement the guidelines, or that they were unaware who else had taken the approach on board and were reluctant to be the first to do so. HCPs' difficulties with guidelines have been illustrated elsewhere: research from the Netherlands^35^ and the USA^25^ ^26^ has shown that HCPs were unsure what guidelines existed and as a result, made conservative assumptions about what sharing was permitted. Thus, more detailed guidelines are not always the answer—especially as they will always allow room for professional judgement, and may lead to inaction, for example, if the particulars of a certain situation are different to the ones outlined.^36^

Although only some participants had considered taking a familial approach to confidentiality, participants were more consistent in taking a relational approach to autonomy.^37^ ^38^ According to Gilbar,^37^ a HCP who is faced with a disclosure dilemma and is taking this relational approach would not only consider clinical factors (eg, whether the risk in question is severe, likely and has an available intervention), but also the effect of disclosure on family relationships. Gilbar also argues that if taking seriously this last criterion, HCPs will have discussed “various aspects of the patient's familial relationships…[and] have close knowledge” of their nature (p391). In our study, participants saw it as an important part of their role to highlight familial implications of any test result. They also considered family relationships when thinking about ways that information might be shared, such as via intermediaries. But, ultimately, they thought patients should share the information.

### Implications for practice and research

We would argue that a familial approach to confidentiality could be taken as the norm—that is, familial genetic information could be shared unless there are good reasons not to—where a result in one person might be relevant to others. Nevertheless, some important issues relating to participants' concerns would need to be addressed and clarified before engaging a familial approach:

- Further research should explore whether separating personal and familial genetic information is possible in cases where there is concern about breaching confidentiality, and in what situations sharing the latter could lead to inference of a patient's identity.
- We recommend that any responsibility HCPs have to relatives be bounded—that is, their responsibility to certain relatives should not imply a duty of care or a duty to warn to all possible at-risk relatives. Having a default position of sharing familial genetic information would then change practice only in narrow circumstances (ie, where the known relative of a patient faces harm and/or could benefit from an intervention). Resource-light ways to contact relatives not yet referred to a genetics service would need to be identified, such as using existing databases and registers to find their contact details, or those of their GP, sending tailored letters directly, and making follow-up phone calls.^20–24^
- We reiterate the JCMG recommendation that HCPs broach with patients from the earliest opportunity (eg, before the test) whether and how genetic information might be shared with family members. This is especially important since our previous research showed that patients want HCPs to tell them at the outset about the possibility of such sharing.^16^

Since participants considered a relational approach to autonomy to be standard practice, and since some had taken a familial approach to confidentiality in some cases, taking the familial approach as default may simply be an extension of these existing practices. HCPs sharing familial genetic information could be more protective of patient privacy than the family-mediated approach: patients in one US study illustrated this point, arguing that HCPs can be trusted to make a distinction between familial information (eg, the genetic mutation) and personal clinical information and share accordingly. Relatives tasked with sharing might not always do so and might, as a result, reveal patient identities.^39^ Indeed, in several published interventions about hereditary cancers, HCPs have safeguarded individual privacy when writing to relatives on a patient's behalf, by simply saying that a member of the family had been found to have an inherited tendency to develop cancer, without initially revealing the gene mutation or specific diagnosis.^20–24^ In one of these interventions, clinicians asked relatives if they thought their privacy was thereby invaded, and notably, they did not.^24^

### Strengths and weaknesses

Our study is the first to explore familial confidentiality and HCPs' responsibilities to relatives. The findings support our systematic review:^16^ participants identified several reasons for not acting on a perceived responsibility to relatives. The study builds on previous work that has interrogated the familial approach, but is novel in that it has explored views about the approach using empirical methods.

There were 11 genetics services from which no HCPs took part. Nevertheless, our findings are still transferable: we have provided enough detail about our research context to enable other genetics services within and outside the UK to determine the extent to which the findings apply to their setting.^40^ One limitation is that some discussions were around hypothetical issues (eg, asking what participants would do if resources and infrastructure were in place). Our wider research is building on this limitation by using longitudinal research to identify ethical issues as they are experienced in practice.

## Conclusions

Overall, this study makes an important and timely contribution to what is known about confidentiality and information-sharing practices at a time when whole-genome analyses are being offered to increasing numbers of patients, in the UK and worldwide. More patients will now be identified as at risk of a heritable condition, and more HCPs will be tasked with communicating this information to patients. We found that participants consider the familial approach to confidentiality difficult to implement in practice. Further research should explore with HCPs whether they consider the barriers they identified surmountable and should explore ways to facilitate the appropriate and secure sharing of familial genetic information to benefit families in practice.
